# Degradable Pseudo Conjugated Polymer Nanoparticles with NIR‐II Photothermal Effect and Cationic Quaternary Phosphonium Structural Bacteriostasis for Anti‐Infection Therapy

**DOI:** 10.1002/advs.202200732

**Published:** 2022-03-27

**Authors:** Huiling Zhou, Dongsheng Tang, Xiaoxu Kang, Haitao Yuan, Yingjie Yu, Xiaolu Xiong, Nier Wu, Fangzhou Chen, Xing Wang, Haihua Xiao, Dongsheng Zhou

**Affiliations:** ^1^ Beijing National Laboratory for Molecular Sciences Key Laboratory of Polymer Physics and Chemistry Institute of Chemistry Chinese Academy of Sciences Beijing 100190 P. R. China; ^2^ University of Chinese Academy of Sciences Beijing 100049 P. R. China; ^3^ Beijing Advanced Innovation Center for Soft Matter Science and Engineering Beijing Laboratory of Biomedical Materials Beijing University of Chemical Technology Beijing 100029 P. R. China; ^4^ State Key Laboratory of Organic‐Inorganic Composites Beijing Laboratory of Biomedical Materials Beijing University of Chemical Technology Beijing 100029 P. R. China; ^5^ State Key Laboratory of Pathogen and Biosecurity Beijing Institute of Microbiology and Epidemiology Beijing 100071 P. R. China

**Keywords:** anti‐infectieon therapy, cationic quaternary phosphonium structural bacteriostasis, nanoparticle, photothermal antibacterial effect, pseudo conjugated polymer

## Abstract

Photothermal therapy based on conjugated polymers represents a promising antibacterial strategy but still possesses notable limitations. Herein, degradable pseudo conjugated polymers (PCPs) containing photothermal molecular backbones and reactive oxygen species (ROS)‐sensitive thioketal bonds are designed. Triphenylphosphine (PPh_3_) is introduced into PCPs to generate phosphonium‐based PCPs (pPCPs), which further assembled with hyaluronic acid into pPCP nanoparticles (pPCP‐NPs). pPCP‐NPs with quaternary phosphonium cations selectively anchor on and destroy bacterial cell membranes through electrostatic action. Under 1064 nm laser irradiation, pPCP‐NPs (pPCP‐NPs/+L) produce near‐infrared‐II (NIR‐II) photothermal antibacterial effect, thereby killing bacteria in a sustained manner. pPCP‐NPs are readily degraded upon ROS abundant at infection sites, therefore exhibiting enough biosafety. pPCP‐NPs/+L display an almost 100% bacterial inhibition rate in vitro and resultin a nearly complete recovery of bacteria‐induced mouse wounds. A further metabolomics analysis denotes that pPCP‐NPs/+L work in a concerted way to induce bacterial DNA damage, inhibit bacterial carbon/nitrogen utilization and amino acid/nucleotide synthesis. Taken together, degradable pPCP‐NPs with both NIR‐II photothermal effect and cationic phosphonium structural bacteriostasis provide a new avenue for antibiotics‐alternative anti‐infection therapy.

## Graphical abstract

pPCP nanoparticles (pPCP‐NPs) were developed to combine near‐infrared‐II (NIR‐II) photothermal effect with cationic phosphonium structural bacteriostasis. Under 1064 nm laser irradiation, pPCP‐NPs (pPCP‐NPs/+L) produce NIR‐II photothermal antibacterial effect, thereby killing bacteria in a sustained manner. Furthermore, the phosphonium structural bacteriostasis could destruct bacterial membrane. This combinational strategy provides a new avenue for antibiotics‐alternative anti‐infection therapy.

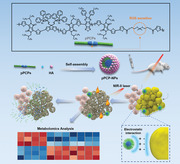

## 1 Introduction

The wide use of antibiotics for clinical treatment of bacterial infections has resulted in the accumulation and dissemination of bacterial antibiotic resistance worldwide especially in hospital settings.^[^ ^1^ ^]^ To cope with this situation, researchers have developed various antibiotics‐alternative strategies such as photothermal therapy,^[^ ^2^ ^]^ photodynamic therapy,^[^ ^3^ ^]^ sonodynamic therapy,^[^ ^4^ ^]^ cationic polymers,^[^ ^5^ ^]^ and stereochemical chemical structures.^[^ ^6^ ^]^ Among them, photothermal therapy is rather promising, because photothermal agents can generate mild heat under external laser irradiation, which steadily kills bacteria via destroying cell membrane permeability, interfering with cell metabolism, and denaturing bacterial proteins. In addition, photothermal therapy is noninvasive and spatiotemporally controllable, which is conducive to the clinical application.^[^ ^7^ ^]^

The photothermal materials can be classified as inorganic nanomaterials and organic conjugated polymers (CPs).^[^ ^8^ ^]^ These photothermal antibacterial materials still have notable limitations. First, inorganic materials such as quantum dots,^[^ ^9^ ^]^ metal‐based nanoparticles/clusters,^[^ ^10^ ^]^ and upconversion nanoparticles^[^ ^11^ ^]^ are nondegradable, which result in long‐term toxicity; organic CPs generally have improved biocompatibility compared to inorganic materials, but they are also nondegradable which brings with long‐term safety concerns. Moreover, it is still challenging to develop degradable CPs with higher safety. Second, the excitation wavelengths for most photothermal agents are in near‐infrared region I (NIR‐I: 650–950 nm), which has a lower ability to penetrate into deep infectious sites to kill deeply seeded bacteria compared to NIR‐II (1000–1700 nm).^[^ ^12^ ^]^ Third, the temperature generated by photothermal conversion should be less than 55 °C,^[^ ^13^ ^]^ which causes minimal body injury and effective bacterial destruction.^[^ ^14^ ^]^ Taken together, it is of great significance to develop organic polymers whichcould be degraded in an expected manner, produce NIR‐II photothermal effect, and be preferably collaborated with other antibacterial strategies.

Quaternary ammonium or phosphonium salts can destroy bacterial cell integrity through electrostatic interaction with negatively charged bacterial cell membranes and have been widely used as antimicrobial agents.^[^ ^15^ ^]^ Quaternary phosphonium salts differ from quaternary ammonium salts by the replacement of N atoms with P atoms. Since P atoms have larger ionic radius and stronger polarization than N atoms have, quaternary phosphonium salts show a higher affinity toward the negatively charged bacterial surface, hence exhibiting better antibacterial effect.^[^ ^16^ ^]^ However, it has been shown in recent years that solely relying on these compounds is highly prone to result in bacterial resistance.^[^ ^17^ ^]^ Therefore, it is meaningful to develop a synergistic antibacterial strategy which combines degradable organic photothermal polymers with cationic phosphonium salts.

**Scheme 1:**
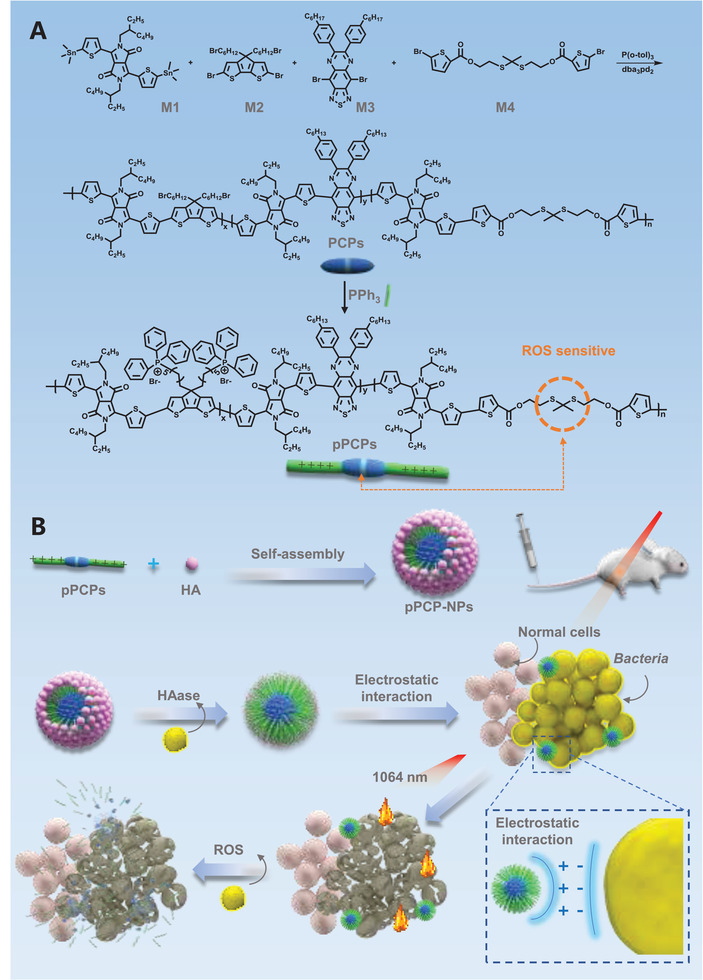
Schematic illustration showing the design of pPCP‐NPs for antibacterial therapy. A) Chemical structure of ROS sensitive NIR‐II photothermal phosphonium‐containing degradable conjugated polymers (pPCPs). B) Negatively charged hyaluronic acid (HA) as the shell of pPCPs formed through electrostatic interaction to form the core–shell nanoparticles pPCP‐NPs. After intravenous injection, pPCP‐NPs were effectively accumulated in the infected site in the mouse model. HA was degradable under the HAase and then pPCPs were released. pPCPs then anchored to bacterial membrane by electrostatic interaction. Under 1064 nm (NIR‐II) laser irradiation, pPCPs generated heat, resulting in bacterial cell death.

Herein, we design degradable pseudo conjugated polymers (PCPs) for achieving effective antibacterial therapy (**Scheme** **1**). PCPs were characterized by the presence of photothermal molecular backbones and reactive oxygen species (ROS)‐sensitive thioketal bonds in the main chain. Triphenylphosphine (PPh_3_) was subsequently introduced into PCPs through Wittig reaction to generate phosphorated PCPs (pPCPs) with quaternary phosphonium cations in the side chains. The cationic PCPs were further used to assemble with negatively charged hyaluronic acid (HA) to form phosphonium‐based pseudo conjugated polymer nanoparticles (pPCP‐NPs). Under 1064 nm laser irradiation, pPCP‐NPs could selectively target bacterial cells with excellent antibacterial efficacy in vitro and in vivo. Compared to the previous study,^[^ ^18^ ^]^ this work has the following two technical breakthroughs: i) pseudo conjugated pPCP‐NPs containing abundant thioketal bonds were readily degraded upon ROS that was abundant at infection sites,^[^ ^19^ ^]^ therefore exhibiting excellent biocompatibility in vitro and in vivo. ii) pPCP‐NPs/+L could achieve deep tissue penetration, thereby guaranteeing an enhanced photothermal antibacterial effect. Taken together, pPCP‐NPs/+L represented an effective antibacterial strategy that combine NIR‐II photothermal antibacterial effect with cationic phosphonium structural bacteriostasis, providing a new avenue for antibiotics‐alternative anti‐infection therapy.

## 2 Results and Discussions

### 2.1 Synthesis and Characterization of PCPs

PCPs were synthesized (Scheme 1A) via polymerization of three commercially available monomers (bistin‐containing monomer M1, bisbromine‐containing monomer M2, and bisbromine‐containing monomer M3) together with the ROS‐sensitive bisbromine‐containing monomer M4 (Figure S1, Supporting Information).^[^ ^20^ ^]^ Thereby, PCPs were obtained with numerous ROS‐sensitive thioketal bonds and photothermal polymeric backbone in the polymer main chain. PPh_3_ was subsequently introduced into PCPs through Wittig reaction^[^ ^21^ ^]^ to generate pPCPs with quaternary phosphonium cations in the side chain (Figure S2, Supporting Information). In order to reduce the toxicity and side effects of positively charged quaternary phosphonium salts, the negatively charged biocompatible HA was adopted to coat as the shell of pPCPs through electrostatic interactions to form the core–shell nanoparticles pPCP‐NPs (Scheme 1B).

**Figure 1:**
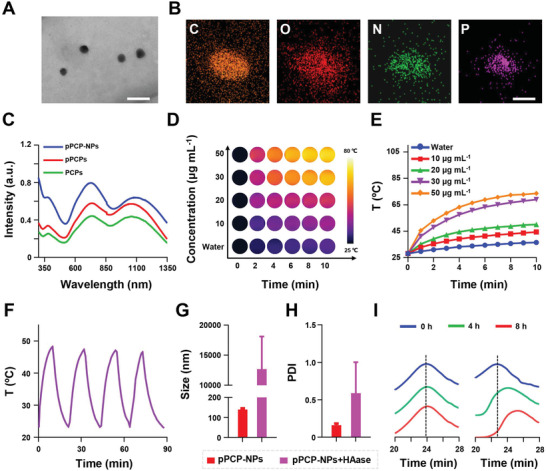
Characterization of pPCP‐NPs. A) TEM images of pPCP‐NPs. Scale bar = 100 nm. B) STEM and EDXS pictures of pPCP‐NPs. Scale bar = 500 nm. C) UV–vis–NIR absorption spectras of PCPs and pPCPs. D) Thermal imaging and E) the corresponding heating curves of pPCP‐NPs. F) Photothermal stability of pPCP‐NPs. Average particle size (G) and particle size distribution (H) of pPCP‐NPs in the presence or absence of HAase measured by dynamic light scattering (DLS). I) GPC curves of CPs (left) and PCPs (right) incubated with 10 × 10^−3^ m H_2_O_2_ for 0, 4, and 8 h respectively.

Subsequently, we adopted the FDA‐approved biocompatible amphiphilic polyethylene glycol‐polyglycolide (PEG‐PLGA) as the shell to assemble with PCPs to form another kind of core–shell nanoparticles PCP‐NPs, which only contain photothermal agents (Scheme S1, Supporting Information). PCP‐NPs were used as a control for pPCP‐NPs in this study. As shown by transmission electron microscope (TEM) (**Figure** **1**A and Figure S3, Supporting Information), the average sizes of PCP‐NPs and pPCP‐NPs were 39.3 ± 4.9 and 40.8 ± 8.5 nm, respectively. Both of them had regular shape and narrow size distribution. As shown by scanning transmission electron microscope (STEM) coupled with energy‐dispersive X‐ray spectroscopy (EDXS) (Figure 1B), pPCP‐NPs contained the key elements such as C, O, N, and P, further confirming the successful conjugation of PPh_3_ to PCPs.

As shown by ultraviolet (UV)–vis–NIR absorption spectras (Figure 1C), PCPs or pPCPs in dichloromethane (DCM) had two major absorption peaks at 734 and 1060 nm, while pPCP‐NPs in water had two corresponding major peaks at 734 and 1119 nm with a small shift of 55 nm. These results showed that pPCP‐NPs had an absorption extended in NIR‐II region. To explore the photothermal properties, pPCP‐NPs at 10–50 µg mL^–1^ were irradiated by a 1064 nm laser at 1 W cm^−2^ for 10 min, followed by recording the time–temperature curves (Figure 1D and E). Results showed that as the concentration of pPCP‐NPs increased, the heating process became faster (Figure 1E). Specifically, for pPCP‐NPs at 50 µg mL^−1^, the temperature rose rapidly to 65 °C after only 10 min of laser irradiation. Furthermore, Such photothermal performance is well maintained after four testing cycles (Figure 1F), indicating a stable NIR‐II photothermal effect of pPCP‐NPs. What is more, the photothermal conversion efficiency (*η*)^[^ ^22^ ^]^ of pPCP‐NPs was calculated as 51.5%, which was higher than that of the majority of previously reported NIR‐II photothermal materials such as FePS_3_ nanosheets (43.3%)^[^ ^23^ ^]^ and SPNΙ‐II (43.4%).^[^ ^24^ ^]^

Subsequently, to test the responsiveness of pPCP‐NPs to hyaluronidase (HAase),^[^ ^25^ ^]^ pPCP‐NPs were incubated with HAase at 750 µg mL^–1^. The results showed that the average particle sizes shifted from 140 to 12 678 nm (Figure 1G), while the particle size distributions shifted from 0.137 to 0.583 (Figure 1H). Thus, we conclude that HA at the shell of pPCP‐NPs could be degraded by HAase, hence leading to the exposure of the core of pPCP‐NPs that contain quaternary phosphonium salts.

To test the ROS responsiveness of PCPs, we synthesized conjugated polymers (CPs) without ROS‐sensitive thioketal bonds (Scheme S2, Supporting Information) beside PCPs with thioketal bonds (Scheme 1A). CPs or PCPs were incubated with 10 × 10^−3^ m of H_2_O_2_ at different time points (0, 4, and 8 h). Gel permeation chromatography (GPC) curves (Figure 1I) showed that the molecular weight of PCPs decreased from 28.8K to 5.3 K as the incubation time increased, indicating the degradation of PCPs in a time‐dependent manner. On the contrary, no obvious change in the molecular weight was observed for CPs in presence of ROS, denoting limited degradation of CPs.

### 2.2 In Vitro Antibacterial Efficacy

**Figure 2:**
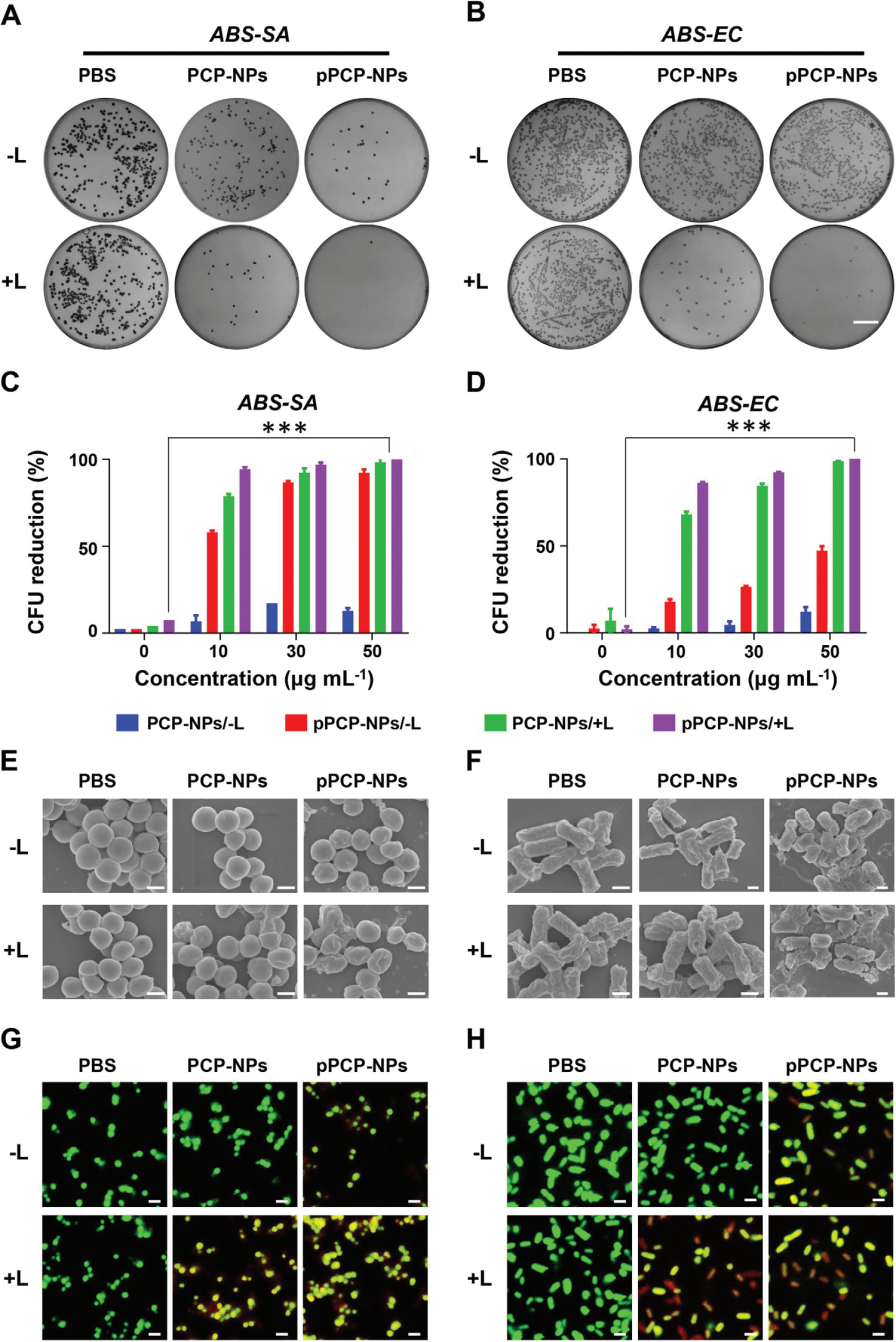
In vitro antibacterial effect against ABS bacteria. A,B) Bacterial CFU formation (scale bar = 2 cm) and C,D) counts on solid medium after treatment by PCP‐NPs or pPCP‐NPs in the presence (+L) or absence (−L) of 1064 nm laser irradiation. SEM (E and F, Scale bar = 0.5 µm) and CLSM (G and H, Scale bar = 2 µm) photographs of bacterial cells after different treatments. Green fluorescence (DMAO: *E*_x_/*E*_m_: 503/530 nm) and red fluorescence (EthD‐III: *E*_x_/*E*_m_: 530/620 nm). PCP‐NPs, pPCP‐NPs: 50 µg mL^–1^.

**Figure 3:**
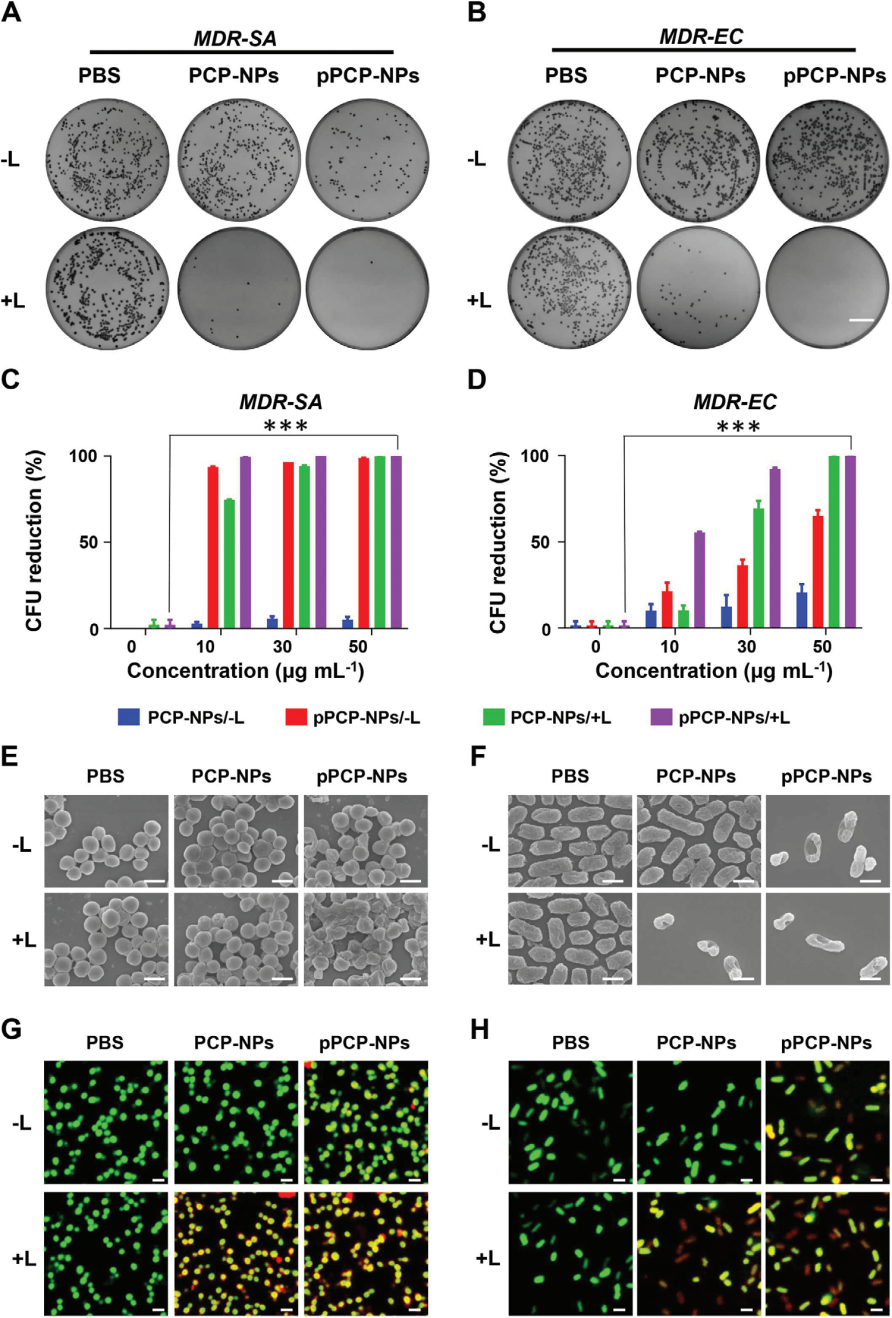
In vitro antibacterial effect against MDR bacteria. A,B) Bacterial CFU formation (Scale bar = 2 cm) and C,D) counts on solid medium after treatment by PCP‐NPs or pPCP‐NPs in the presence (+L) or absence (−L) of 1064 nm laser irradiation. E,F) SEM pictures (Scale bar = 1 µm) and G,H) CLSM photographs of bacterial cells after different treatments (Scale bar = 2 µm). Green fluorescence (DMAO: *E*_x_/*E*_m_: 503/530 nm) and red fluorescence (EthD‐III: *E*_x_/*E*_m_: 530/620 nm).PCP‐NPs, pPCP‐NPs: 50 µg mL^–1^.

The in vitro antibacterial efficacy was characterized against two representative antibiotic‐susceptible (ABS) bacteria *ABS‐SA* (*Staphylococcus aureus* American Type Culture Collectio (ATCC） 25923) and *ABS‐EC* (*Escherichia coli* ATCC 25923) (**Figure** **2**), which were the reference bacterial strains susceptible to almost all the commonly used antibiotics. A series of meaningful results could be summarized as follows: First, almost no bacterial colony‐forming units (CFUs) could be observed on solid medium after the treatment of pPCP‐NPs/+L (NIR‐II photothermal antibacterial effect plus quaternary phosphonium cationic bacteriostasis); by contrast, either PCP‐NPs/+L (NIR‐II photothermal antibacterial effect alone) or pPCP‐NPs/−L (quaternary phosphonium cationic bacteriostasis alone) could not completely inhibit bacterial growth on solid medium (Figure 2A,B). Second, the inhibition rate of pPCP‐NPs/+L against *ABS‐SA* or *ABS‐EC* reached nearly 100%, which were higher than that of PCP‐NPs/+L and pPCP‐NPs/−L; for pPCP‐NPs/+L, PCP‐NPs/+L, and pPCP‐NPs/−L, the inhibition rates enhanced significantly with the increase of nanoparticle concentrations (Figure 2C,D). Third, as shown by scanning electron microscope (SEM） (Figure 2E,F), the morphological damage effect of pPCP‐NPs/+L against bacterial cells were higher than that of PCP‐NPs/+L or pPCP‐NPs/−L. Finally, the dead/live staining results obtained by confocal laser scanning microscopy (CLSM) (Figure 2G,H) showed that bacterial cells treated with pPCP‐NPs/+L were almost entirely stained with green and red while those treated with PCP‐NPs/+L or pPCP‐NPs/−L were merely stained with green; these observations were consistent with the fact that the green stain could label bacterial cells with intact or damaged membranes whereas the red stain would only penetrate bacteria cells with damaged membranes. Notably, we also applied all the above in vitro antibacterial experiments to two clinical multidrug resistant (MDR) isolates *MDR‐EC (E. coli* 2364) and *MDR‐SA* (*S. aureus* 120016‐1) from Chinese public hospitals, and essentially the same results were obtained (**Figure** **3**). Taken together, pPCP‐NPs/+L combined NIR‐II photothermal effect with cationic phosphonium mediated bacteriostasis, thereby possessing nearly 100% inhibition rate against *S. aureus* and *E. coli*.

### 2.3 A Metabonomics View of Antibacterial Mechanism

**Figure 4:**
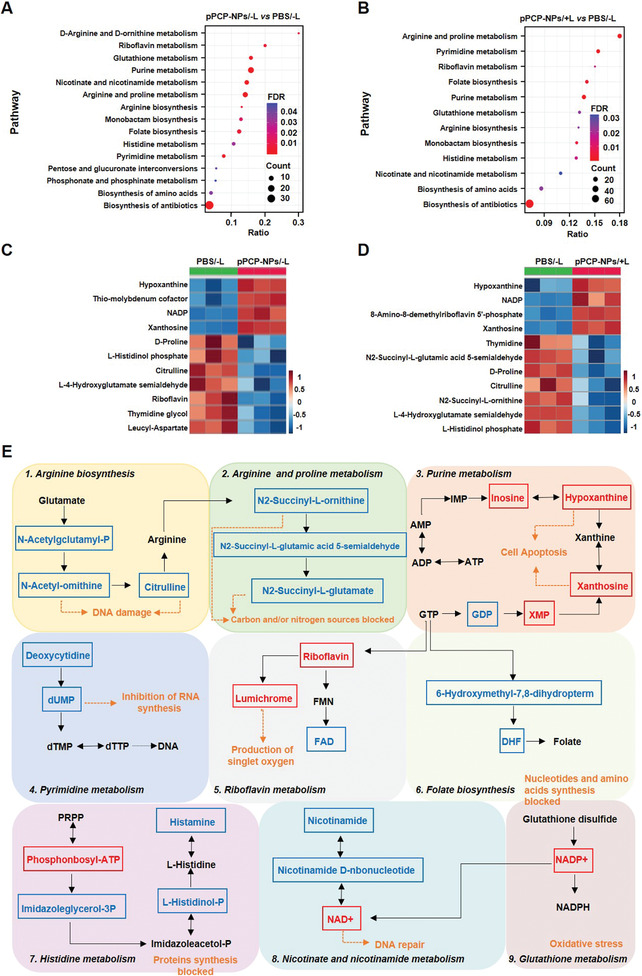
Metabonomics analysis of *MDR‐SA* after different treatments in vitro. KEGG analysis of the major differentially regulated metabolic pathways for A) the pPCP‐NPs/−L group or B) the pPCP‐NPs/+L group as compared to the PBS/−L group. Heatmaps of the major differentially regulated metabolites for C) the pPCP‐NPs/−L group or D) the pPCP‐NPs/+L group as compared to the PBS/−L group. E) Schematic overview of the major affected bacterial metabolic pathways after pPCP‐NPs/+L treatment. Red represented upregulation and blue stood for downregulation.

A metabonomics analysis of differentially regulated metabolites and further functional enrichment of these metabolites were performed for *MDR‐SA* after different treatments in vitro. First, the principal component analysis and cluster analysis showed that the phosphate buffered saline (PBS）/−L, pPCP‐NPs/−L, and pPCP‐NPs/+L groups were distributed at three distant quadrants, indicating the presence of obvious differences among various groups but parallelism within each group (Figure S4A, Supporting Information). Second, compared to PBS/−L, there were 1227 upregulated and 313 downregulated metabolites in bacteria treated with pPCP‐NPs/−L and 1267 upregulated and 510 downregulated metabolites in bacteria treated with pPCP‐NPs/+L; there were 91 upregulated and 175 downregulated metabolites in bacteria treated with pPCP‐NPs/+L relative to pPCP‐NPs/−L (Figure S4B–D, Supporting Information). Third, Kyoto Encyclopedia of Genes and Genomes (KEGG） analysis was conducted to find out the major metabolic pathways related to antibacterial effect; pPCP‐NPs/−L principally affected the metabolisms of riboflavin, glutathione, nicotinate, nicotinamide, purine, and pyrimidine, while pPCP‐NPs/+L mainly affected not only the above pathways but also the metabolisms of arginine and proline (**Figure** **4**A,B and Figure S4E, Supporting Information). Fourth, we presented the different profiles of major differentially regulated metabolites for the pPCP‐NPs/−L and pPCP‐NPs/+L groups as compared to the PBS/−L group; hypoxanthine, nicotinamide adenine dinucleotide phosphate (NADP), and xanthosine were upregulated while citrulline, riboflavin, and thymine glycol were downregulated in the pPCP‐NPs/−L group; hypoxanthine and xanthosine were upregulated while N2‐succinyl‐l‐ornithine, citrulline, l‐histidinol phosphate, and d‐proline were downregulated in the pPCP‐NPs/+L group (Figure 4C,D and Figure S4F, Supporting Information). The upregulation of hypoxanthine would induce cell death and production of ROS,^[^ ^26^ ^]^ while the upregulation of xanthosine would have a negative effect on cell proliferation.^[^ ^27^ ^]^ Finally, the above major differentially regulated metabolites were assigned in the corresponding metabolic pathways after pPCP‐NPs/+L treatment. Taken together, pPCP‐NPs/+L induced bacterial DNA damage and cell apoptosis, destroyed bacterial cell redox balance, and inhibited bacterial carbon/nitrogen utilization and amino acid/nucleotide synthesis (Figure 4E).

### 2.4 Antibacterial Therapy Efficacy in Mouse Model

Human normal epithelial cell line IOSE was chosen to test the potential toxicity of pPCP‐NPs and the results showed that the survival rate of IOSE‐80 cells was 76.3% after coincubation with pPCP‐NPs at 50 µg mL^–1^ for 8 h (Figure S5, Supporting Information), indicating that pPCP‐NPs had no significant cytotoxicity to normal human cells.

**Figure 5:**
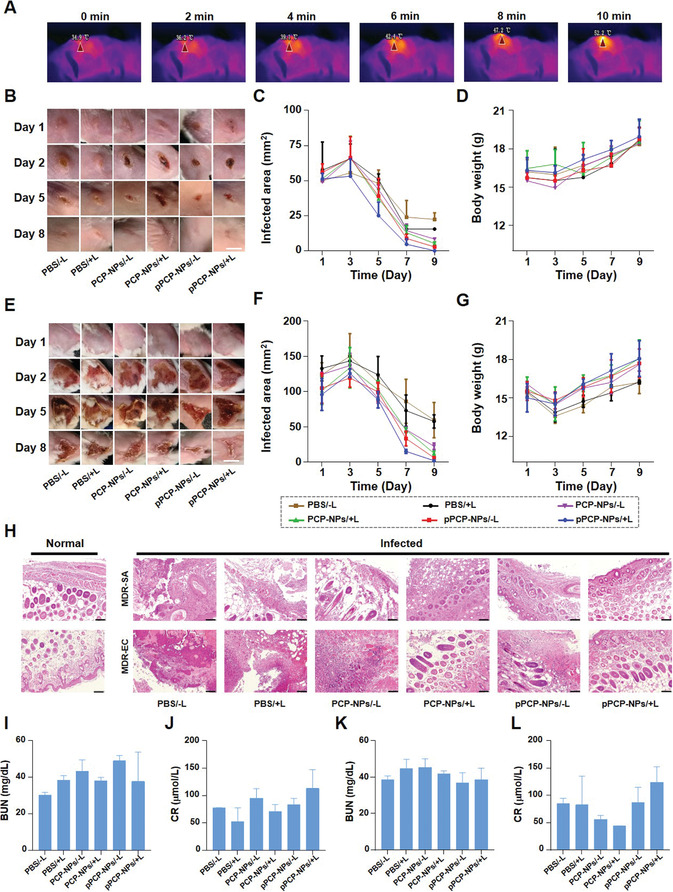
In vivo antibacterial effect against *MDR‐SA* and *MDR‐EC*. A) In vivo temperature increase for pPCP‐NPs/+L. Representative gross appearance of wounds infected by B) *MDR‐SA* and E) *MDR‐EC* after different treatments. Scale bar = 5 mm. Mean infected areas of wounds infected by C) *MDR‐SA* and *F) MDR‐EC* after different treatments. Mean body weights of mice infected by D) *MDR‐SA* and G) *MDR‐EC* after different treatments. Scale bar = 5 mm. H) Representative photos of hematoxylin and eosin (H&E) staining of normal skin and wounds. Scale bar = 100 µm. G) Detection of serum biochemical indicators mice infected by I,J) *MDR‐SA* and K,L) *MDR‐EC* after different treatments.

The mouse models with *MDR‐SA*‐ or *MDR‐EC*‐infected wounds were established to evaluate in vivo antibacterial activity. First, to study the distribution of pPCP‐NPs in vivo, Cy7.5 as a fluorescent probe was encapsulated in pPCP‐NPs and then pPCP‐NPs/Cy7.5 were intravenously injected into mice; pPCP‐NPs/Cy7.5 could reach the infected area at 2 h postinjection and maintained for at least 48 h (Figure S6, Supporting Information). Second, upon 10 minutes 1064 nm laser irradiation at 1 W cm^−2^, the in vivo temperature increased up to 52.2 °C, which was suitable for photothermal therapy and had little adverse effect on surrounding normal tissues (**Figure** **5**A). Third, for *MDR‐SA*‐infected wounds, pPCP‐NPs/+L led to a nearly 100% decrease in the infected area (i.e., almost completely recovery) at 8 d post‐treatment, and this value was much higher than that of all the other treatment groups (Figure 5B,C); the body weights of *MDR‐SA*‐infected mice decreased at 3 d and then increased gradually for all the treatment groups, denoting that the health status of mice was getting better (Figure 5D). Furthermore, similar results were observed for *MDR‐EC*‐induced wound infections (Figure 5E–G). Fourth, H&E staining showed that the pPCP‐NPs/+L group exhibited nearly normal histological status while serious dermal structure destruction and infectious skin lesions were observed for all the other treatment groups (Figure 5H). Fifth, slight increase in the levels of blood urea nitrogen (BUN) and creatinine (CREA) were observed for both *MDR‐SA* and *MDR‐EC* infection models (Figure 5I–L). Finally, H&E staining showed no obvious pathological toxicity in heart, liver, spleen, lung, and kidney of uninfected mice for all the treatment groups (Figure S7, Supporting Information). Taken together, pPCP‐NPs/+L exhibited excellent in vivo antibacterial efficacy against *S. aureus* and *E. coli* with favorable biosafety.

## 3 Conclusion

In summary, we reported here a novel kind of degradable PCPs for preparing pPCP‐NPs containing ROS‐sensitive thioketal bonds, NIR‐II photothermal agents, and quaternary phosphonium cations for effective antibacterial therapy. The degradation of pPCP‐NPs was triggered by ROS, which was abundant at infection sites. The antibacterial effect can be attributed to the NIR‐II photothermal agents cooperates with the cationic bacteriostasis resulted from the quaternary phosphonium salts. The in vitro inhibition rates of pPCP‐NPs/+L against *S. aureus* and *E. coli* reached nearly 100%. A metabolomics analysis denoted that pPCP‐NPs/+L induced bacterial DNA damage and cell apoptosis, destroyed bacterial cell redox balance, and inhibited bacterial carbon/nitrogen utilization and amino acid/nucleotide synthesis. In a mouse model of skin wound infections caused by MDR‐*S. aureus* and *E. coli*, pPCP‐NPs/+L resulted in a nearly complete recovery of infection wounds. pPCP‐NPs/+L had a broad‐spectrum antibacterial effect against Gram‐positive and Gram‐negative bacteria in vitro and in vivo with favorable biosafety. Degradable pPCP‐NPs that combined NIR‐II photothermal antibacterial effect with phosphonium cationic bacteriostasis would have an immense potential for clinical application as an antibiotics‐alternative strategy of anti‐infection therapy.

## 4 Experimental Section

### Materials

5‐Bromo‐2‐thiophenecarboxylic acid, PPh_3_, and tris (dibenzylideneacetone) dipalladium (dba_3_pd_2_) (CAS: 51364‐51‐3) were purchased from Aladdin (Shanghai, China). 1‐(3‐Dimethylaminopropyl)‐3‐ethylcarbodiimide was purchased from Sigma‐Aldrich (St. Louis, MO, USA). Tri(*o*‐tolyl)phosphine (P(o‐tol)_3_) (CAS: 6163‐58‐2) and Cy7.5 were purchased from Energy Chemical (Shanghai, China). 2,5‐Bis(2‐ethylhexyl)‐3,6‐bis(5‐(trimethylstannyl)thiophen‐2‐yl)pyrrolo[3,4‐c]pyrrole‐1,4(2H,5H)‐dione (DPP26‐2Sn), 2,6‐Dibromo‐4,4‐bis(6‐bromohexyl)‐4H‐cyclopenta[2,1‐b:3,4‐b’]dithiophene (DTCC6Br4), and 4,9‐dibromo‐6,7‐bis(4‐hexylphenyl)‐[1,2,5]thiadiazolo[3,4‐g]quinoxaline (DPTQ6‐2Br) were purchased from Alfa (Zhengzhou, China). 3‐(4,5‐Dimethylthiazol‐2‐yl)‐2,5‐diphenyltetrazolium bromide (MTT) and sodium dodecyl sulfate (SDS) were purchased from beyotime (Shanghai, China). Cell culture vessels were purchased from Corning (Corning, NY, USA). Roswell Park Memorial Institute ‐1640 (RPMI‐1640) medium, fetal bovine serum (FBS), 0.25% trypsin‐Ethylene Diamine Tetraacetic Acid（EDTA), and penicillin/streptomycin (P/S) were purchased from Gran Island (NY, USA). HA and PEG‐PLGA were purchased from Sigma‐Aldrich (St. Louis, MO, USA). Tryptone soybean broth (TSB) and Tryptic Soy Agar (TSA) were purchased from Solarbio (Beijing, China). Methanol, toluene, acetonitrile, glutaraldehyde, tetrahydrofuran, and ethanol were purchased from Concord (Tianjin, China). Viability/cytotoxicity assay for Bacteria Live and Dead Cells kit was purchased from Biorigin (Beijing, China).

### Synthesis of M4

2,2′‐(Propane‐2,2‐diylbis(sulfanediyl)bis(ethan‐1‐ol) (1.98 g, 10.10 mmol), 5‐bromothiophene‐2‐carboxylic acid (5.22 g, 25.25 mmol), 1‐ethyl‐3‐(3‐dimethylaminopropyl) carbodiimide hydrochloride (4.84 g, 25.25 mmol), and 4‐dimethylaminopyridine (3.08 g, 25.25 mmol) were dissolved in 100 mL N,N‐Dimethylformamide（DMF） and incubated at room temperature for 24 h. The reaction system was poured into 500  mL water, then extracted with 100 mL ethyl acetate for three times, and separated by column chromatography to obtain a white solid M4 with a yield of 68%. ^1^H NMR (400 MHz, CDCl_3_) *δ* 7.55 (d, 2H), 7.06 (d, 4H), 4.43 (t, 4H), 2.97 (t, 4H), 1.65 (s, 6H).

### Synthesis of CPs

M1 (80 mg, 0.094 mmol), M2 (31.11 mg, 0.047 mmol), M3 (31.35 mg, 0.047 mmol), P(o‐tol)_3_ (2.39 mg, 0.078 mmol), and dba_3_pd_2_ (1.79 mg, 0.0019 mmol) were dissolved in 5 mL of degassed toluene, protected by nitrogen, and reacted at 120 °C for 3 h. The reaction solution was dropped into 500 mL of anhydrous methanol, stood for 30 min, filtered to obtain dark green precipitation, and finally dried to obtain CPs.

### Synthesis of PCPs

M1 (80 mg, 0.094 mmol), M2 (21.80 mg, 0.033 mmol), M3 (31.35 mg, 0.047 mmol), M4 (8.10 mg, 0.014 mmol), P(o‐tol)_3_ (2.39 mg, 0.078 mmol), dba_3_pd_2_ (1.79 mg, 0.0019 mmol) were dissolved in 5 mL of degassed toluene, protected by nitrogen gas, and incubated at 120 °C for 3 h, and then the reaction solution was dropped into 500 mL of anhydrous methanol, stood for 30  min, filtered to obtain dark green precipitation, and finally dried to obtain PCPs.

### Synthesis of pPCPs

PCPs (25 mg) and PPh_3_ (30 mg) were dissolved in a mixture of 10 mL toluene and 15 mL acetonitrile, protected by nitrogen gas, and stirred under reflux at 90 °C. After 48 h of reaction, the reaction solution was further handled as above to obtain pPCPs.

### Preparation of PCP‐NPs

PEG‐PLGA (10.0 mg) and PCPs (1.0 mg) were completely dissolved with 1 mL of tetrahydrofuran (THF) by bath sonication. The mixture was added dropwise into deionized water (9.0 mL) under continuous sonication at a power of 80 W for 3 min, and THF in the solution was then removed by dialysis with a dialysis bag (molecular weight cutoff, 7000 Da) for 24 h, obtaining the PCP‐NPs solution.

### Preparation of pPCP‐NPs

HA (10.0 mg) was completely dissolved with 9 mL of deionized water and pPCPs (1.0 mg) were completely dissolved with 1 mL of THF. The pPCP‐NPs solution was added dropwise into the HA solution (9.0  mL) under continuous sonication at a power of 80 W for 3 min, and THF was removed as above, obtaining the pPCP‐NPs solution. All nanoparticle solutions were stored at 4 °C for further use.

### Preparation of pPCP‐NPs labeled with Cy7.5

HA (10.0 mg) was completely dissolved in 9 mL of deionized water. pPCPs (1.0 mg) and Cy7.5 (1.0 mg) were completely dissolved in 1 mL of THF. The pPCPs+Cy7.5 solution (1 mL) was added dropwise into the HA solution (9.0 mL) under continuous sonication at a power of 80 W for 3 min, and then THF was removed as above, obtaining the Cy7.5‐labeled pPCP‐NPs solution.

### Characterization of Polymers or Nanoparticles

^1^H NMR spectra was measured by a 400 MHz NMR spectrometer (Bruker) at room temperature. GPC was performed using a GPC‐1515 instrument (USA) [Column type: Styragel HT6E*2, HT2, Column length: 7.8*30 mm, Detector: 2414, pump 1515, Test temperature: 40 ℃, flow: 1 mL min^−1^, eluent: THF]. UV–Vis–NIR spectra were recorded using a spectrophotometer (TU‐1901). The photothermal performance was measured by an infrared thermal camera (FOTRIC 225s). Size and zeta potential measurements were conducted on a Zetasizer (Nano ZS, UK). TEM images were recorded by and Hitachi HT‐7700 TEM (Japan), while STEM images were recorded by JEOL JEM‐2100F STEM (USA). MTT assay was conducted using a Microplate reader (SpectraMax). The small animal imaging was performed by an In Vivo Imaging System (IVIS, Perkin Elmer).

### In Vitro Photothermal Assay

For photothermal conversion performance study, the deionized water solution of pPCP‐NPs was continuously exposed to 1064 nm laser irradiation at 1 W cm^–2^ for 10 min. The temperature was monitored using an FOTRIC 225s infrared thermal camera. For *η* calculation, 1 mL of pPCP‐NPs solution (25 µg mL^–1^) in quartz cell was irradiated by a 1064 nm laser at 1 W cm^–2^ until the temperature of the solution stopped rising. The heating and cooling curves of the solution were recorded by FOTRIC 225s. The *η* value was calculated via Equation (1) and (2), where *m* was the solvent mass and *C* was the solvent specific heat capacity; *K*_s_ was the solution time constant, which was calculated by the fitting curve of time versus −ln*θ*, obtained from the cooling curve; Δ*T*_max_ and Δ*T*_solvent_ were the maximum temperature variation of pPCP‐NPs solution and solvent, respectively; *I* was the laser power; *A_*λ*_* was the absorbance of pPCP‐NPs solution at 1064 nm.

(1) $$hA\;=\frac{mC}{K_{s}}\;$$

(2) $$\eta \;=\frac{hA\left(\Delta T_{\max}-\Delta T_{\mathrm{solvent}}\right)}{I\left(1-10^{-A\lambda }\right)}\;$$

For photothermal stability study, 1 mL of pPCP‐NPs solution (25 µg mL^−1^) was irradiated by a 1064 nm laser (1 W cm^−2^) for four ON/OFF cycles. The temperature variation of the solution was recorded by FOTRIC 225s.

### Bacterial Cultivation

*S. aureu*s and *E. coli* were cultured in TSB medium in a shaking incubator (200 rpm) at 37 °C and harvested at the logarithmic growth phase by centrifugation at 4500 g for 5 min. After washing with PBS for three times, the bacteria were resuspended in PBS for further use. The concentration of bacteria was monitored by measuring the optical density at 600 nm (OD_600_) using a UV spectrophotometer.

### In Vitro Antibacterial Experiments

The bacteria were diluted to 1 × 10^5^ to 1 × 10^6^ CFU mL^−1^ and then treated with different nanomaterial formulations for 4 h at 37 °C on a shaking incubator (200 rpm). The PBS/+L, PCP‐NPs/+L, and pPCP‐NPs/+L treatment groups were separately irradiated with a 1064 nm laser (1 W cm^−2^, 10 min). Each bacterial suspension was serially diluted 1 × 10^4^‐fold with sterile water, and 100 µL of each diluted bacterial suspension was spread on TSA medium, followed by overnight incubation at 37 °C. The inhibition rate was calculated according to the following equation, where *C* was CFUs of the PCP‐NPs/−L or PCP‐NPs/+L or pPCP‐NPs/−L or pPCP‐NPs/+L group, and *C*_0_ was CFUs of the control PBS/−L group.

(3) $$\mathrm{Inhibition}\mathrm{rate}=\frac{C_{0}-C}{C_{0}}\times 100\%$$

### Bacterial Morphology Assay

Bacterial suspensions (1 × 10^5^ to 1 × 10^6^ CFU mL^−1^) were subjected to different treatments and incubated for 4 h at 37 °C. For SEM, the bacterial suspensions were centrifuged at 4500 g for 10 min and then washed three times with PBS, and the collected bacterial cells were fixed with 2.5% glutaraldehyde overnight at 4 °C. After washing with PBS three times, 5 µL of bacterial suspension was dropped onto silicon slices. Bacteria cells were dehydrated through sequential treatments of 30%, 50%, 70%, 80%, 90%, 95%, and 100% ethanol for 30 min. Bacterial morphology was observed by SEM with a JSM‐6700F electron microscope.

### Bacterial Live/Dead Staining Assay

The viability of bacteria cells was qualitatively assessed using a Live/Dead BacLight Bacterial Viability Kit. Bacteria suspensions at 1 × 10^5^ to 1 × 10^6^ CFU mL^−1^ were subjective for different treatments for 4 h and collected by centrifugation at 10 000 × *g* for 3 min. After washing with 0.85% NaCl, bacteria cells were stained with 1 µL of 100 × green‐fluorescent nucleic acid stain (DMAO) and red‐fluorescent nucleic acid stain (EthD‐III) for 15 min. The activity of bacterial cells was monitored via CLSM (ZEISS LSM 880).

### In Vitro Cytotoxicity Assay

IOSE cells were seeded in 96‐well plates (1×10^4^ cells per well) and incubated with RPMI1640 supplemented with 10% FBS (150 µL) at 37 °C for 24 h. Then, the cells were treated for 8 h with PCP‐NPs/−L or pPCP‐NPs/−L at 10–50 µg mL^−1^. Thereafter, the cellular viability was assessed via an MTT colorimetric assay. In brief, MTT reagent (10 µL of a 5 µg mL^–1^ solution in PBS buffer) was added to each well and the plates were further allowed to incubate with cells for another 4 h. Acidified SDS solution was then added (100 µL per well) and the plates were kept in the dark for an additional 12 h. Measurements of absorbance were subsequently made with a Bio‐Rad plate reader (Spectra Max M3) at 570 nm (peak absorbance) and subtracted at 650 nm (background absorbance).

### In Vivo Biodistribution Imaging

The 6‐ to 8‐week‐old female BALB/c mice purchased from Beijing Charles River Company were used for all the animal experiments. pPCP‐NPs (corresponding to 0.3 mg mL^−1^ pPCPs) labeled with Cy7.5 (1.5 mg kg^−1^) was intravenously injected into uninfected mice. Ex vivo biodistribution imaging of various organs was collected by collected by IVIS (Spectrum （CT））, PerkinElmer, *E*_x_/*E*_m_ = 780 nm/810 nm). All animal experiment protocols were reviewed and approved by the Institutional Animal Care and Use Committee of Beijing Institute of Microbiology and Epidemiology (permit number IACUC‐DWZX‐2021‐060).

### Mouse Skin Wound Infection Models

The right sides of the backs of mice were shaved and then subcutaneously inoculated with bacterial suspensions (1 × 10^6^ to 1 × 10^7^ CFU mL^−1^, 100 µL) to establish skin wound infections.

### In Vivo Antibacterial Experiments

Mice at 24 h postinfection were randomly divided into seven treatment groups: 100 µL of each of PBS/−L, PBS/+L, PCP‐NPs/−L, PCP‐NPs/+L, pPCP‐NPs/−L, and pPCP‐NPs/+L were intravenously injected twice (days 1 and 4). pPCP‐NPs/−L used herein corresponded to 0.3 mg mL^−1^ pPCPs. Laser irradiation as above was applied to the PCP‐NPs/+L and pPCP‐NPs/+L group after each injection. The infected areas, and the damaged areas, and the body weights were measured. The infected skin tissues after different treatments were collected and fixed in 4% fixative solution, subjected to H&E staining, and finally examined using a digital microscope.

### In Vivo Biosafety Assessments

The blood samples of infected mice after different treatments were collected by retro‐orbital bleeds and centrifuged at 800 × *g* for 20 min, and the serum samples were analyzed for two renal function markers BUN and CREA. Various organs of *MDR‐SA*‐infected mice after the above treatments were subjected to H&E staining histological analysis as above.

### Statistical Analyses

Data were calculated and processed as mean ± standard deviation (SD). Comparison between groups were analyzed with student t test and one‐way analysis of variance. Graphs were plotted and statistical difference analyses were conducted using GraphPad Prism 7.0 (*p < 0.05, **p < 0.01, and ***p < 0.001).

## Conflict of Interest

The authors declare no conflict of interest.

## Supporting information

Supporting Information

## Data Availability

Research data are not shared.
