# Supplementary material for: Degradable Pseudo Conjugated Polymer Nanoparticles with NIR‐II Photothermal Effect and Cationic Quaternary Phosphonium Structural Bacteriostasis for Anti‐Infection Therapy
Source: Adv Sci (Weinh). 2022 Mar 27;9(16):2200732. doi: 10.1002/advs.202200732 (PMC9165483; doi:10.1002/advs.202200732)
Supplement: Supplementary file 1 — Supporting Information [file ADVS-9-2200732-s001.pdf]

ADVANCED SCIENCE
Open Access

Supporting Information

for *Adv. Sci.*, DOI 10.1002/advs.202200732

Degradable Pseudo Conjugated Polymer Nanoparticles with NIR-II Photothermal Effect and Cationic Quaternary Phosphonium Structural Bacteriostasis for Anti-Infection Therapy

Huiling Zhou, Dongsheng Tang, Xiaoxu Kang, Haitao Yuan, Yingjie Yu, Xiaolu Xiong, Nier Wu, Fangzhou Chen, Xing Wang, Haihua Xiao* and Dongsheng Zhou**

Supporting Information

Degradable Pseudo Conjugated Polymer Nanoparticles with NIR-II Photothermal Effect and Cationic Quaternary Phosphonium Structural Bacteriostasis for Anti-infection Therapy

Huiling Zhou, Dongsheng Tang, Xiaoxu Kang, Haitao Yuan, Yingjie Yu, Xiaolu Xiong, Nier Wu, Fangzhou Chen, Xing Wang, Haihua Xiao*, Dongsheng Zhou**

H. Zhou, D. Tang, Prof. H. Xiao

Beijing National Laboratory for Molecular Sciences, Key Laboratory of Polymer Physics and Chemistry, Institute of Chemistry, Chinese Academy of Sciences, Beijing, 100190, P. R. China.

E-mails: hhxiao@iccas.ac.cn.

H. Zhou, D. Tang, H. Yuan, Prof. H. Xiao

University of Chinese Academy of Sciences, Beijing 100049, P. R. China

X. Kang, Prof. X. Wang

Beijing Advanced Innovation Center for Soft Matter Science and Engineering, Beijing Laboratory of Biomedical Materials, Beijing University of Chemical Technology, Beijing 100029, P. R. China

E-mail: wangxing@mail.buct.edu.cn

Dr. Y. Yu

State Key Laboratory of Organic-Inorganic Composites; Beijing Laboratory of Biomedical Materials; Beijing University of Chemical Technology, Beijing 100029, P.R. China

X. Xiong, N. Wu, F. Chen, Prof. D. Zhou
State Key Laboratory of Pathogen and Biosecurity, Beijing Institute of Microbiology and Epidemiology, Beijing 100071, P. R. China
E-mail: zhouds@bmi.ac.cn, dongshengzhou1977@gmail.com

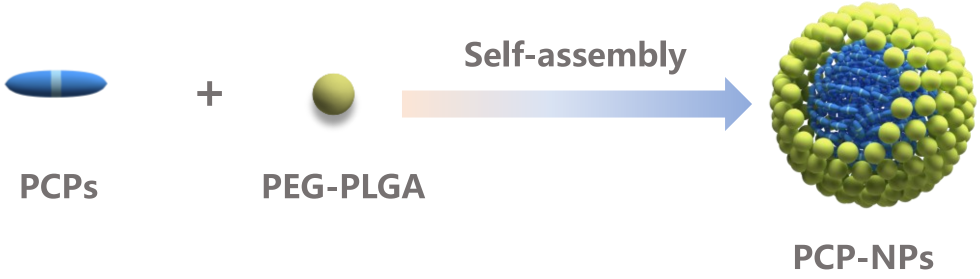

Scheme S1. Schematic illustration showing the fabrication process of PCP-NPs.

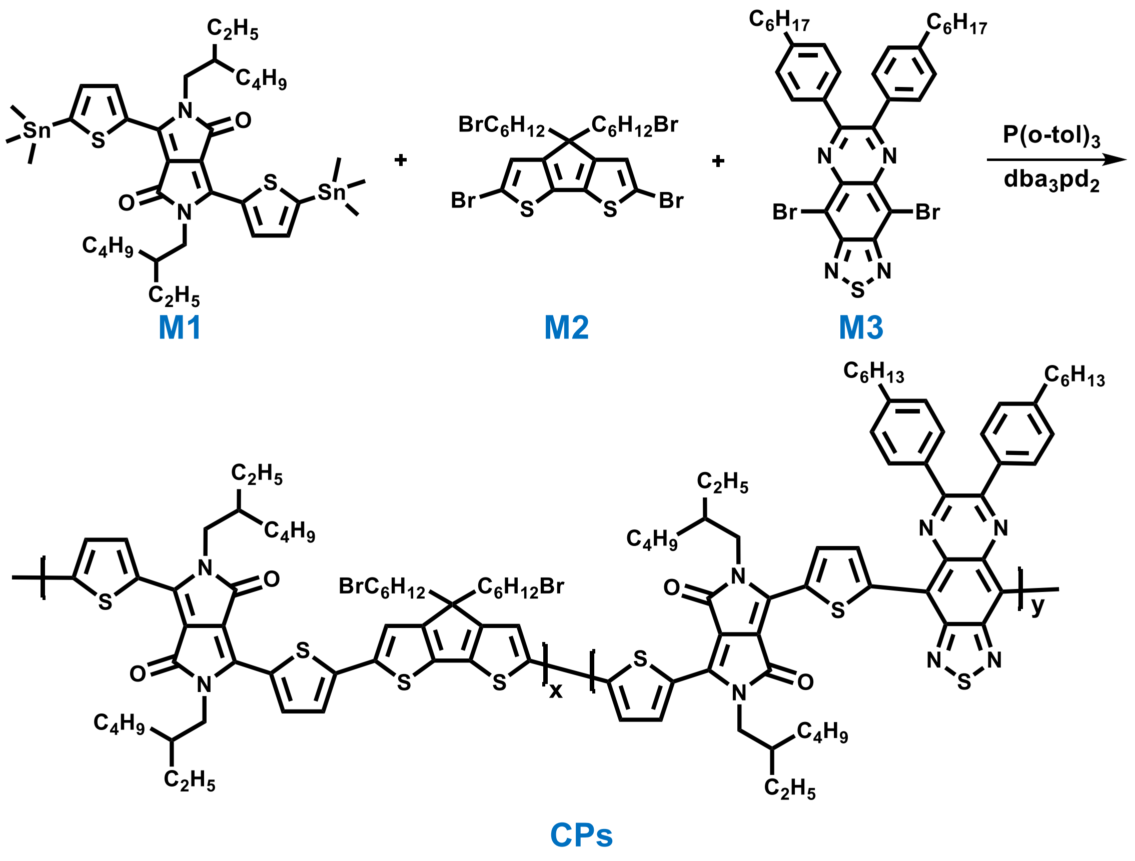

Scheme S2. Synthesis route of CPs without ROS-sensitive bonds.

A

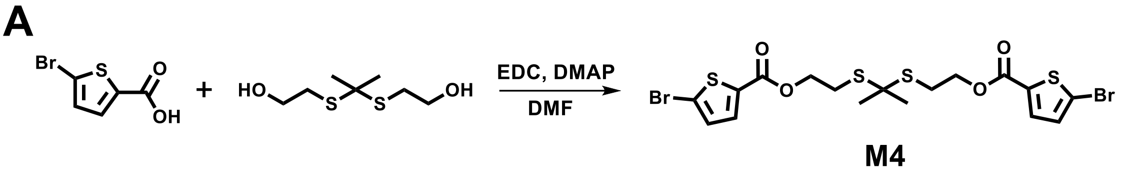

B

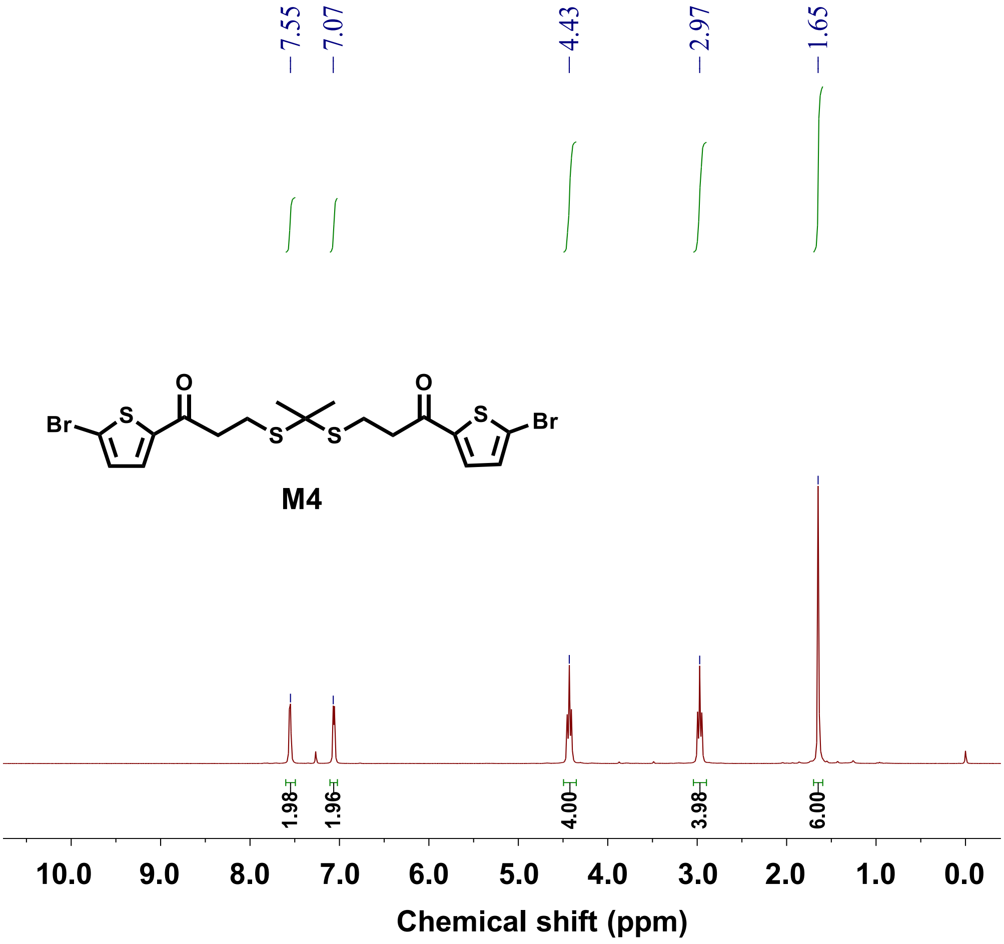

Figure S1. Characterization of M4. A) Synthesis of M4. B) ^{1}H NMR spectrums of M4.

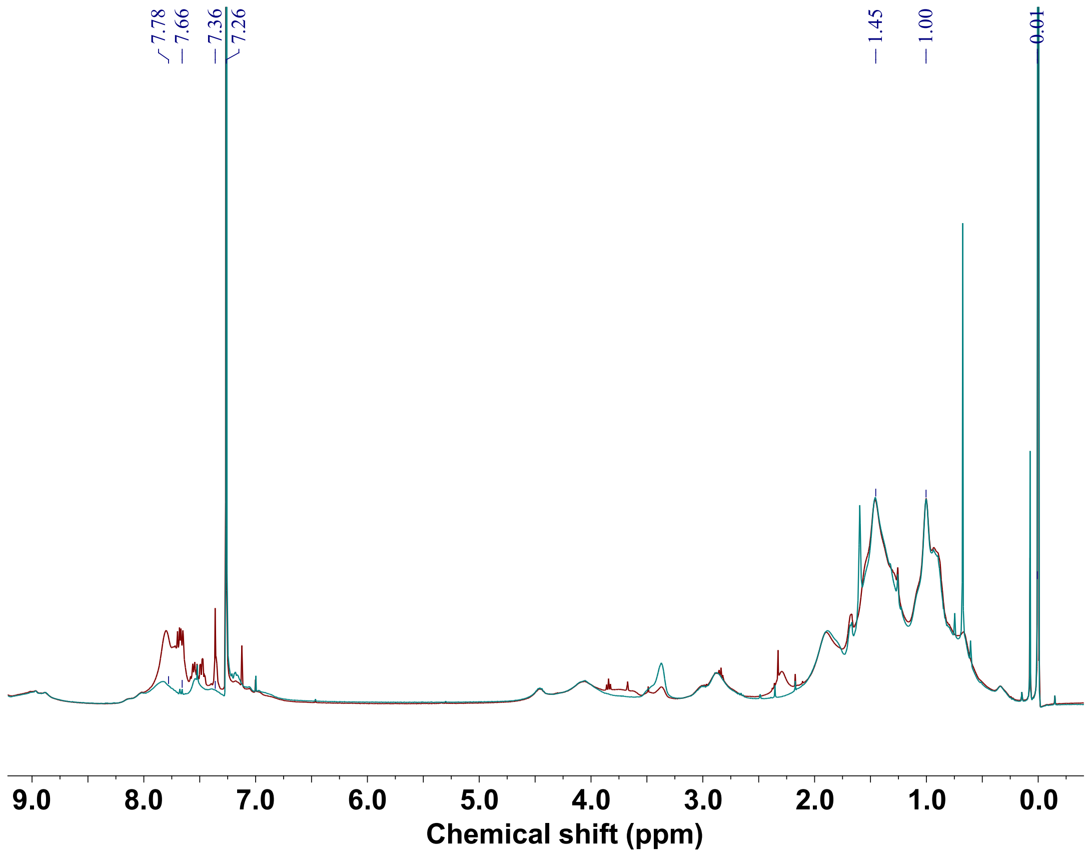

Figure S2. ^{1}H NMR spectrums of PCPs and pPCPs (Red line indicates pPCPs, and blue line stands for PCPs.).

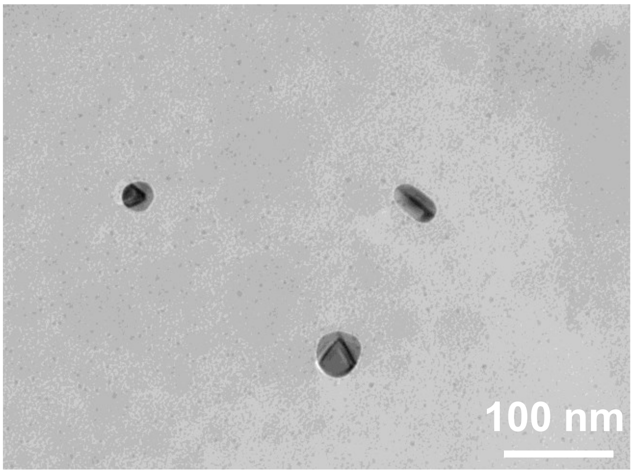

Figure S3. TEM images of PCP-NPs.

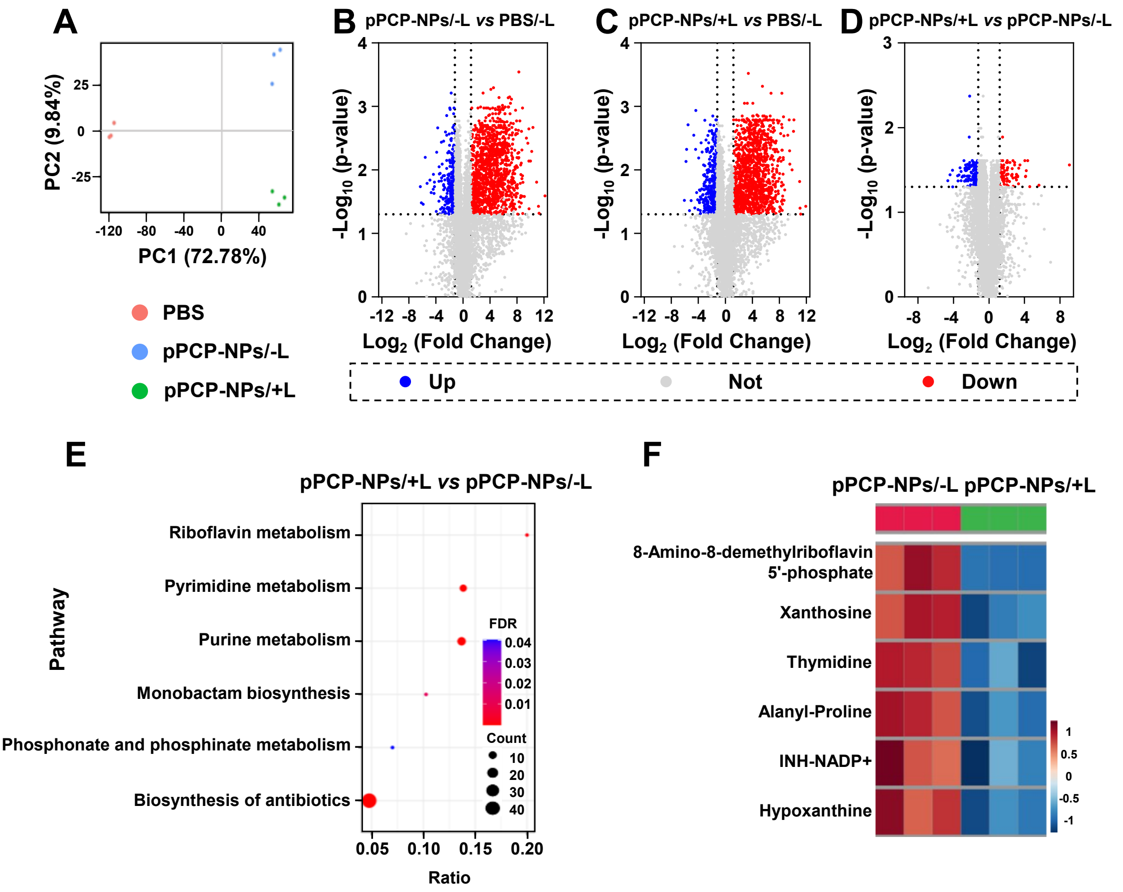

Figure S4. Metabonomics analysis of MDR-SA after various treatments. A) PCA clustering among the three treatment groups PBS/-L, pPCP-NPs/-L, and pPCP-NPs/+L. B, D) Comparison of expression levels of pair-wised metabolites among three treatments groups. Blue color represented down-regulation. Red color represented up-regulation. Gray color stood for no significant change. E) KEGG analysis of metabolic pathways of the pPCP-NPs/+L group compared to pPCP-NPs/-L group. F) Heatmap analysis for identified interested metabolites of pPCP-NPs/+L group as compared to the pPCP-NPs/-L group.

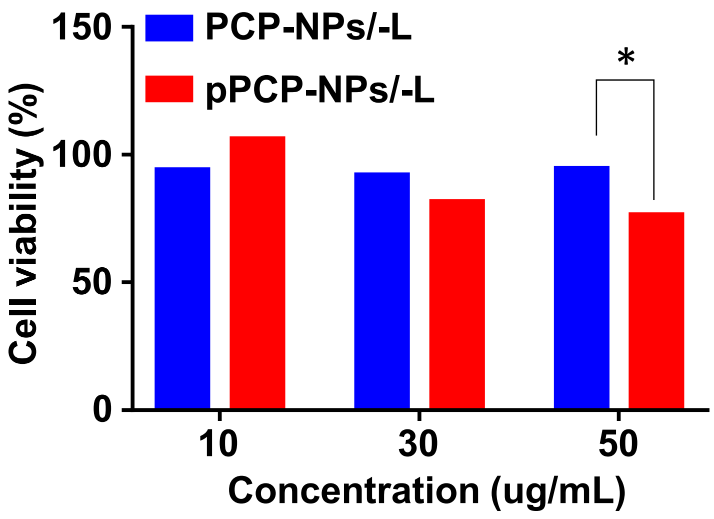

Figure S5. Cytotoxicity of PCP-NPs/-L and pPCP-NPs/-L to human IOSE cells.

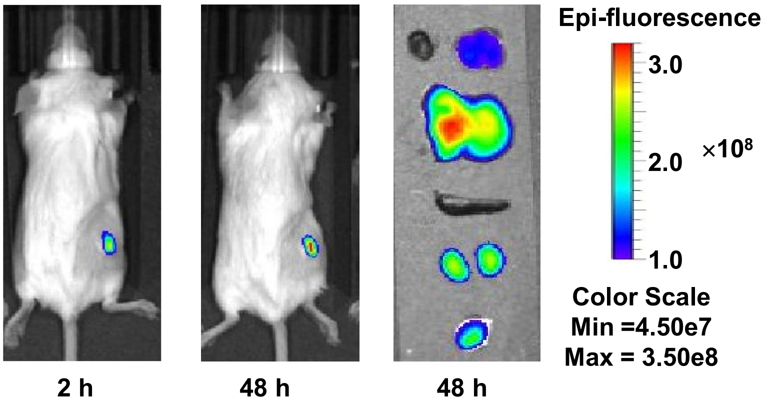

Figure S6. Representative biodistribution study *via* fluorescence imaging of pPCP-NPs in infected skin wounds after intravenous injection.

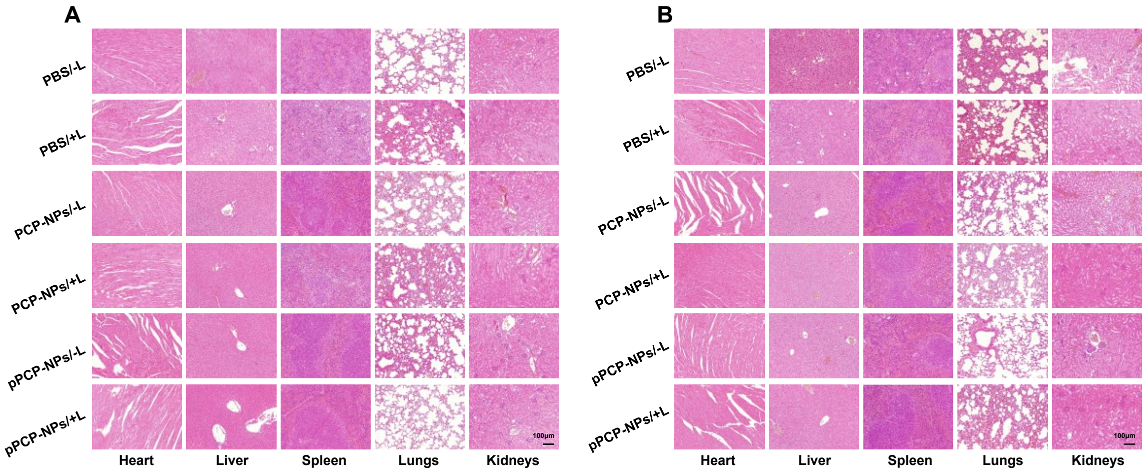

Figure S7. H&E staining of various organs from infected mice at the end of treatment. A) H&E staining of various organs from MDR-SA-infected mice at the end of treatment of PBS/-L, PBS/+L, PCP-NPs/-L, PCP-NPs/+L, pPCP-NPs/-L, and pPCP-NPs/+L. B) H&E staining of various organs from MDR-EC-infected mice at the end of treatment of PBS/-L, PBS/+L, PCP-NPs/-L, PCP-NPs/+L, pPCP-NPs/-L, and pPCP-NPs/+L.
